# Predicting outcomes in patients with exacerbation of COPD requiring mechanical ventilation

**DOI:** 10.1186/s13613-024-01394-z

**Published:** 2024-10-20

**Authors:** Obaid Alzaabi, Emmanuel Guerot, Benjamin Planquette, Jean-Luc Diehl, Thibaud Soumagne

**Affiliations:** 1 Medical Intensive Care Unit, European Hospital of George Pompidou, 20 Rue Leblanc, 75015 Paris, France; 2 Pulmonary Intensive Care Unit, European Hospital of George Pompidou, Paris, France

**Keywords:** COPD, Exacerbation, Outcome, Mechanical ventilation, Mortality

## Abstract

**Background:**

Acute exacerbations of COPD (AECOPD) are common and significantly contribute to mortality in patients with COPD. Prognostic scores can assist clinicians in making tailored decisions to manage AECOPD. In the current study, we therefore aimed to evaluate the performance of the Noninvasive Ventilation Outcomes (NIVO) score, originally designed to assess in-ICU mortality, in predicting 1 year mortality and NIV failure in AECOPD.

**Methods:**

This retrospective study analyzed data from patients hospitalized for AECOPD requiring mechanical ventilation between January 1st, 2018, and December 31st, 2022. Mortality was assessed at the end of ICU stay and 1 year after admission, while NIV failure was defined as intubation or death without intubation.

**Results:**

Among 302 ICU admissions of COPD patients, 190 patients with AECOPD requiring mechanical ventilation were included. Of these, 44 (23%) died in the ICU, 62 out of 184 (34%) failed NIV, and 78 (41%) died within 1 year of admission. Patients who died in ICU or experienced NIV failure had more severe COPD and more impaired blood gas parameters at admission. The NIVO score demonstrated an AUC of 0.68 in predicting 1-year mortality and an AUC of 0.85 in predicting NIV failure. A NIVO score over 7 was associated with higher 1-year mortality and NIV failure (HR of 4.4 [1.8–10.9] and 41.6 [5.6–307.9], respectively).

**Conclusion:**

Beyond predicting in-ICU mortality, the NIVO-score is a reliable tool in predicting 1-year mortality and NIV failure in AECOPD.

**Supplementary Information:**

The online version contains supplementary material available at 10.1186/s13613-024-01394-z.

## Introduction

Chronic obstructive pulmonary disease (COPD) is a leading cause of mortality worldwide and represents a substantial burden [1, 2]. Acute exacerbations of COPD (AECOPD) are common and recognized as the main contributor to mortality in patients with COPD [3]. According to a large study conducted in France, more than 29,000 patients were hospitalized due to AECOPD between 2010 and 2012, resulting in a mortality rate of over 8% during hospitalization and up to 24% in ICU [4]. The implementation of prognostic scoring systems has emerged as a valuable resource, enabling healthcare professionals to make informed decisions and tailor interventions according to the specific needs of individual patients [5]. Amongst these scoring systems, the DECAF score (the Dyspnea, Eosinopenia, Consolidation, Acidemia, and atrial Fibrillation) has been developed to predict mortality in patients admitted for AECOPD, regardless of the presence of acidemia. Its overall performance is good [area under the receiver operating characteristic curve (AUC) ranging from 0.82 to 0.86] but diminishes in patients requiring mechanical ventilation [6]. The Noninvasive Ventilation Outcomes (NIVO) score has been recently developed to predict mortality in patients with AECOPD requiring mechanical ventilation either non-invasive ventilation (NIV) or invasive mechanical ventilation (IMV) [7]. This score relies on six easily quantifiable variables assessed at admission (presence of chest radiograph consolidation, arterial blood pH, level of consciousness as per the Glasgow coma scale, extended Medical Research Council dyspnea scale (eMRCD), time to acidemia and presence of atrial fibrillation) and has been built through a large multicenter population in United Kingdom, including a derivation and a validation cohort. As a result, the NIVO score accurately predicts mortality risk for patients admitted with AECOPD requiring mechanical ventilation (AUC of 0.79). However, this prediction score has only been evaluated for short term mortality [median time to inpatient death of 7 (2–14) days]. One-year survival is also of importance when deciding on the intensity of care for a patient with AECOPD and acute hypercapnic respiratory failure (AHRF) but the ability of the NIVO score to predict mid-term survival has not been evaluated yet.

Furthermore, in case of severe AECOPD with AHRF, current guidelines advocate for NIV use. Indeed, NIV has demonstrated up to 65% reduction in intubation requirement and a significant decrease in mortality rates among patients with COPD and AHRF [8, 9]. The decision to initiate mechanical ventilation is complex, demanding expertise, precise timing, and a thorough understanding of treatment efficacy and success rates. Nevertheless, non-invasive ventilation (NIV) is frequently underutilized or inadequately administered [10–12]. Additionally, in certain cases, the rate of NIV failure can reach up to 70% [6]. Clinicians’ predictions of outcomes can be pessimistic, potentially leading to underutilization [13]. Therefore, early identification of risk of NIV failure is also crucial for patients with AECOPD and AHRF. Several prediction scores exist but the possibility of using a score which predict both mortality and NIV failure may be appealing [14]. The performance of NIVO score in forecasting the likelihood of successful NIV intervention or the risk of endotracheal intubation remains however unexplored [7].

Therefore, the objectives of the current study were primarily to assess the performance of NIVO score to predict 1-year mortality among patients with AECOPD requiring mechanical ventilation and to explore its performance to predict NIV failure.

## Material and method

### Data sources

In this retrospective study, we included patients hospitalized in the Medical Intensive Care Unit in the European Hospital of George Pompidou in Paris—France for an AECOPD between 1st January 2018 and 31st December 2022. Patients were identified by “Programme de Médicalisation des Systèmes d’Information” (PMSI) codes corresponding to French national hospital discharge database which includes hospital diagnoses and medical procedures performed during each stay in French public hospitals (Table S1). Then, data were individually extracted from the medical records.

### Study population

Our study included patients of 40 years of age and older, hospitalized at least one night for an AECOPD requiring mechanical ventilation (either invasive or NIV). PMSI codes based on international classification disease (ICD)-10 were used to select patient with a principal diagnosis (PD) of COPD (suspected or confirmed) or PD of acute respiratory failure, respiratory infection, influenzae, acute heart failure or pneumothorax and a second associated diagnosis (SAD) of COPD (Table S1) [15]. Exacerbation criteria were defined as acute worsening of respiratory symptoms that results in additional therapy and hospitalisation [16]. Patients with other diagnosis than COPD (COVID19, bronchiectasis, asthma) or COPD without exacerbation criteria were excluded. Patients were included only once, based on their first admission, regardless of multiple admissions thereafter. The NIVO score was calculated prior initiation of mechanical ventilation using the six easily quantifiable variables assessed (presence of chest radiograph consolidation, arterial blood pH, level of consciousness as per the Glasgow coma scale, extended Medical Research Council dyspnea scale (eMRCD), time to acidemia, and presence of atrial fibrillation). The NIVO risk score categorizes patients into four risk levels: low-risk (0–2), medium-risk (3–4), high-risk (5–6), and very high-risk (7–9).

### Outcomes

Mortality was assessed at the end of ICU stay (in-ICU mortality) and 1-year after ICU admission among all patients and in a subgroup of patients who were alive at 30 days of admission. NIV failure was defined as intubation or death without intubation as reported previously [17].

### Ethics

According to French Law, retrospective observational database study did not require approval by an ethics committee or informed signed consent from the patients. This work complied to the protection of personal health data and the protection of privacy with the framework of application provided for by article 65-2 of the amended Data Protection Act and the general data protection regulations. The study was designed according to the STROBE guidelines and was approved by the Institutional Review Board of the French learned society for respiratory medicine—Société de Pneumologie de Langue Française (CEPRO 2024-030).

### Statistical analyses

Categorial variables were compared by Chi-square or Fisher tests. Continuous variables were summarized as mean ± standard deviation and compared by Student tests. Performance of NIVO score for predicting mortality (either 1-year or in-ICU mortality) and NIV failure was assessed according to the receiver operating characteristic curve analysis (AUC). In addition, the relation between NIVO score and mortality or NIV failure was evaluated using Kaplan–Meier survival analysis and Log rank test. All statistics were performed using R version 4.3.2.

## Results

**Fig. 1:**
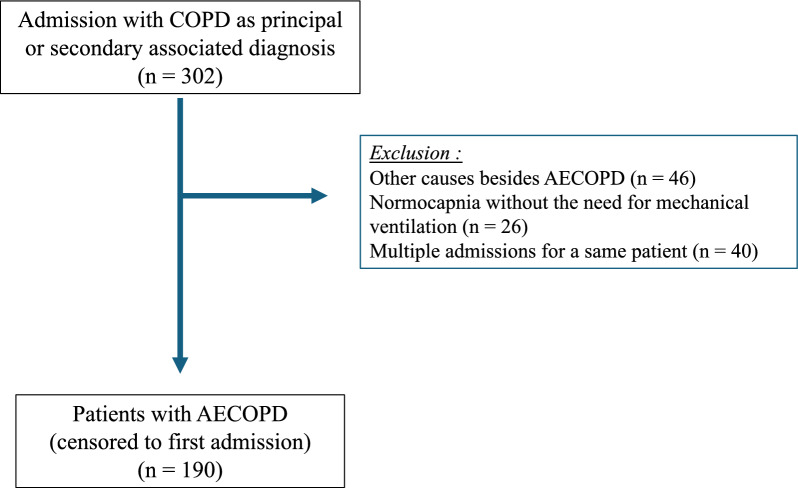
Flow chart of the study

A total of 302 admissions with COPD as principal or secondary associated diagnosis were recorded in the ICU departments at Georges Pompidou European Hospital between January 2018 and December 2022. Out of these, 190 patients met the selection criteria of AECOPD requiring mechanical ventilation, as illustrated in Fig. 1.

The mean age of patients with hypercapnic AECOPD was 73 ± 12 years, including 108 (57%) males. 89 out of 140 patients (64%) severe or very severe COPD (i.e. FEV1 < 50%) and 23% had long-term oxygen therapy (LTOT) before admission. 56% had eMRCD score of 5a or 5b and chest radiograph consolidation was present in 39%.

### NIVO score performance for predicting mortality

**Fig. 2:**
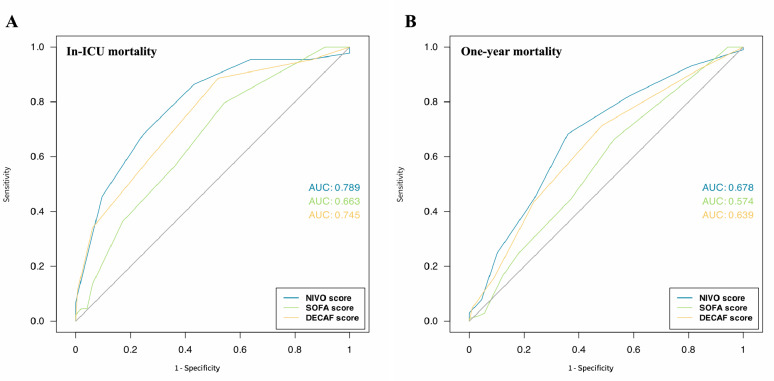
Receiver operating characteristic (ROC) curves of NIVO, DECAF and SOFA scores for in-ICU mortality (**A**) and 1-year mortality (**B**). Area under the ROC curve (AUC) values are also given

**Fig. 3:**
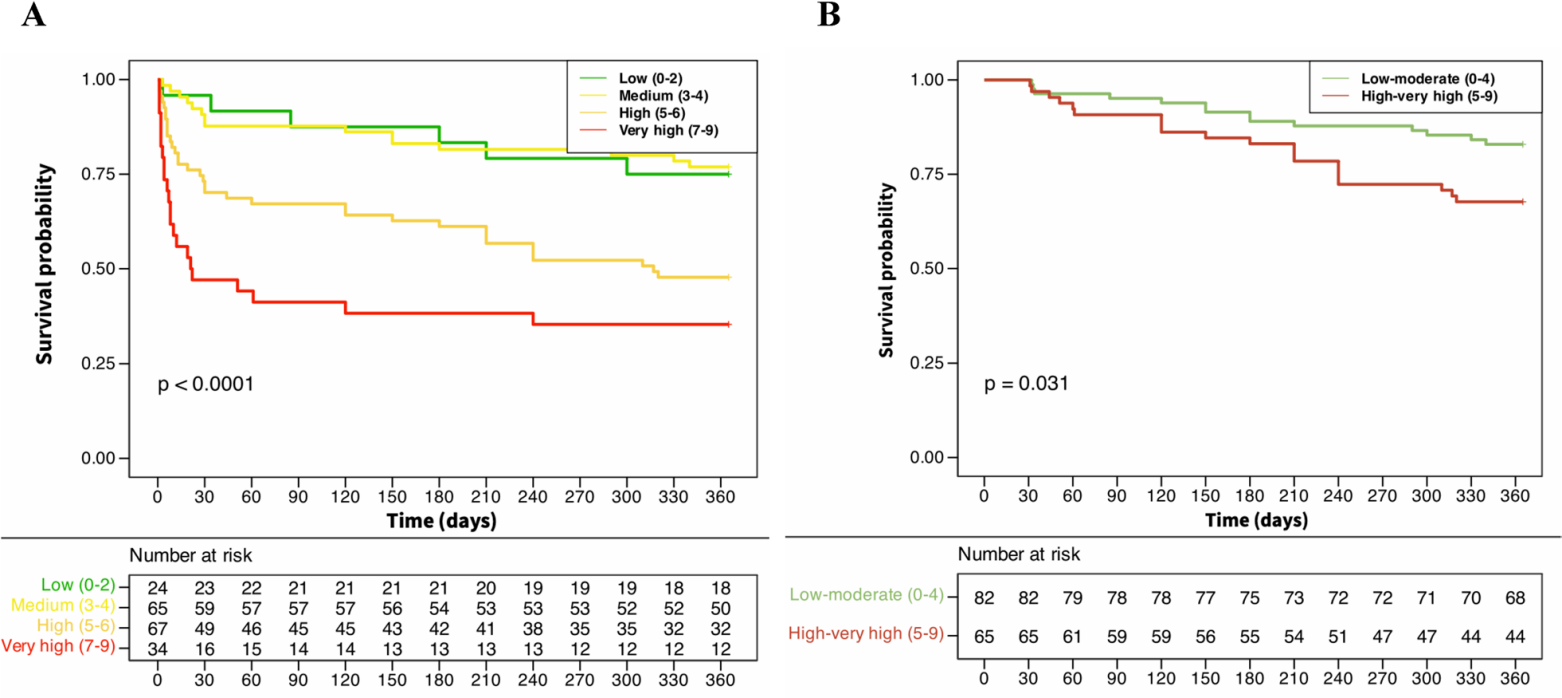
Kaplan–Meier estimates of overall survival in patients with low (0–2), moderate (3–4), high (5–6) and very high risk (7–9) of mortality according to NIVO score (**A**) and among the subgroup of patients (n = 147) who were still alive at day 30 of admission (**B**)

Among included patients, 44 (23%) died in ICU and 78 (41%) died within 1 year of admission. Median (IQR) length of stay in ICU was 6 (4–11) days, median time to in-ICU death was 7 (3–19) days and median follow-up was of 545 (48–1250) days. Patients who died in-ICU had significantly more severe COPD (poorer FEV1 and LTOT requirement), more impaired arterial blood gaze parameters and more frequently chest X-ray consolidation (Table 1). All prognostic scores (SOFA, DECAF and NIVO score) were higher in non-survivors than in survivors (Table 1). Furthermore, performance of NIVO score to predict in-ICU mortality (AUC of 0.79) was higher than that of DECAF or SOFA score (AUC respectively of 0.75 and 0.66) (Fig. 2A). Regarding 1 year mortality, the NIVO-score performed better (AUC 0.68) than the DECAF and the SOFA score (AUC respectively of 0.64 and 0.57) (Fig. 2B). In addition, high and very high NIVO-scores (score of 5–6 and score over 7 respectively) were associated not only with higher in-ICU mortality but also with higher 1 year mortality (HR of 2.6 [1.1–6.2] and 4.4 [1.8–10.9] respectively, plogrank < 0.0001) than low or moderate score (< 5) (Fig. 3A).

**Table 1:** Main characteristics of included patients

	Survivors (in-ICU)	Non-survivors (in-ICU)	P value
	n = 146	n = 44	
Demographics			
Male	83 (56)	25 (56)	1
Age, years	72.2 ± 12.0	76.0 ± 11.3	0.06
FEV_1_, % predicted^a^	48.0 ± 18.8	40.0 ± 13.7	0.007
LTOT	26 (18)	17 (39)	0.007
Previous admission in ICU	63 (43)	31 (70)	0.003
AECOPD in the 12 last months	0.6 ± 1.1	1.1 ± 1.0	< 0.0001
Comorbidities			
Diabetes	30 (21)	8 (18)	0.89
Cardiovascular disease	74 (51)	22 (50)	1
Hypertension	72 (49)	27 (61)	0.21
Chronic kidney disease	29 (20)	9 (21)	1
Chronic liver disease	2 (1)	1 (2)	0.54
At admission			
Chest X-ray consolidation	46 (32)	29 (66)	< 0.0001
Atrial fibrillation	28 (19)	11 (25)	0.53
Glasgow coma score	15 (14–15)	14 (13–15)	0.08
Heart rate, per min	97 ± 22	98 ± 21	0.86
Respiratory rate, per min	26 ± 7	28 ± 8	0.13
Mean blood arterial pressure, mmHg	94 ± 20	91 ± 23	0.49
pH	7.27 ± 0.08	7.24 ± 0.09	0.047
PaO_2_, mmHg	96.4 ± 61.5	82.4 ± 36.6	0.06
PaO_2_/FiO_2_	240 ± 91	192 ± 59	< 0.0001
PaCO_2_, mmHg	69.2 ± 16.6	73.6 ± 20.6	0.20
Time to acidemia > 12 h	141 (97)	42 (95)	0.66
eMRCD5a	43 (29)	14 (32)	0.91
eMRCD5b	27 (19)	40 (98)	< 0.0001
Outcomes and scores			
NIV failure	21 (14)	41 (93)	< 0.0001
SOFA score	3.2 ± 1.8	4.2 ± 2.0	0.002
DECAF	1.7 ± 1.1	2.9 ± 1.3	< 0.0001
NIVO	4.3 ± 1.6	6.1 ± 1.7	< 0.0001

Data are expressed as mean ± SD or median (interquartile range) or n (%). Time to acidaemia > 12 h should be positively scored if: > 12 h have elapsed between arrival at hospital and index episode of acidaemia

*FEV1* forced expiratory volume in 1 s, *LTOT* long-term oxygen therapy, *CVD* cardiovascular diseases, *eMRCD* extended Medical Research Council dyspnea scale, *NIV* noninvasive ventilation, *SOFA score* Sequential Organ Failure Assessment score, *DECAF* dyspnea, eosinopenia, consolidation, acidosis, atrial fibrillation, *NIVO score* Noninvasive Ventilation Outcomes score

^a^Data available for 140 patients

When excluding the 43 patients who died within the 30 days of ICU admission, higher NIVO score at admission still remained associated with higher 1-year mortality (plogrank = 0.031) (Fig. 3B).

### NIVO score performance for predicting NIV failure

At ICU admission, 184 patients received non-invasive ventilation and 6 received invasive mechanical ventilation. Out of these 184 patients, 62 (34%) had NIV-failure with a median failure delay of 12 (2–24) hours. NIV failure resulted in IMV in 38 (61%) patients and ICU-death in 41 (66%) patients.

Patients who had NIV failure had more severe COPD, more impaired arterial blood gaze parameters (PaCO_2_ and PaO_2_/FiO_2_) and Glasgow score at admission (Table 2). Furthermore, NIV failure was associated with higher in-ICU and 1 year mortality.

**Table 2:** Main characteristics of patients with NIV success and NIV failure

	NIV success	NIV failure	P value
	n = 122	n = 62	
Demographics			
Male	64 (52)	41 (66)	0.11
Age, years	72.8 ± 12.7	74.1 ± 10.8	0.47
FEV_1_, % predicted^a^	49.7 ± 18.9	40.9 ± 15.2	0.004
LTOT	22 (18)	21 (34)	0.03
Previous admission in ICU	49 (41)	42 (68)	0.0008
AECOPD in the 12 last months	0.6 ± 1.1	1.1 ± 0.9	0.001
Comorbidities			
Diabetes	25 (20)	11 (18)	0.80
Cardiovascular disease	60 (49)	32 (52)	0.88
Hypertension	61 (50)	34 (55)	0.64
Chronic kidney disease	22 (18)	14 (23)	0.55
Chronic liver disease	2 (2)	1 (2)	1
At admission			
Chest X-ray consolidation	26 (21)	43 (69)	< 0.0001
Atrial fibrillation	25 (20)	14 (23)	0.89
Glasgow coma score	15 (14–15)	14 (13–15)	0.0002
Heart rate, per min	98 ± 21	98 ± 23	0.84
Respiratory rate, per min	26 ± 7	28 ± 8	0.16
Mean blood arterial pressure, mmHg	95 ± 19	93 ± 22	0.69
pH	7.28 ± 0.07	7.23 ± 0.08	0.0005
PaO_2_, mmHg	95.9 ± 61.5	85.0 ± 43.4	0.17
PaO_2_/FiO_2_	244 ± 96	203 ± 60	0.0007
PaCO_2_, mmHg	67.9 ± 16.9	74.7 ± 18.4	0.02
Time to acidemia > 12 h	117 (96)	61 (98)	0.65
eMRCD5a	28 (23)	25 (40)	0.02
eMRCD5b	22 (18)	27 (44)	0.0004
Outcomes and score			
Invasive ventilation (after NIV failure)	0	38 (61)	–
ICU mortality	0	41 (66)	–
One-year mortality	31 (26)	44 (71)	< 0.0001
NIVO score	3.9 ± 1.5	6.2 ± 1.5	< 0.0001

Data are expressed as mean ± SD and median (interquartile range) or n (%). Time to acidaemia > 12 h should be positively scored if: > 12 h have elapsed between arrival at hospital and index episode of acidaemia

*FEV1* forced expiratory volume in 1 s, *LTOT* long-term oxygen therapy, *CVD* cardiovascular diseases, *eMRCD* extended Medical Research Council dyspnea scale, *NIV* noninvasive ventilation, *SOFA score* Sequential Organ Failure Assessment score, *DECAF* dyspnea, eosinopenia, consolidation, acidosis, atrial fibrillation, *NIVO score* Noninvasive Ventilation Outcomes score

^a^Data available in 140 patients

**Fig. 4:**
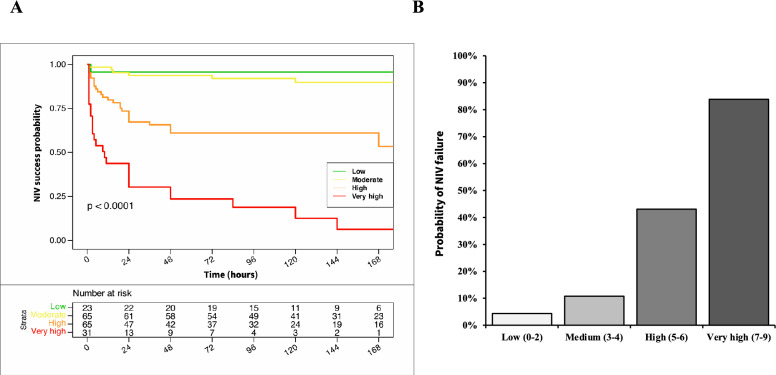
Kaplan–Meier estimates of NIV failure (**A**) and proportion of NIV failure (**B**) in patients with low (0–2), moderate (3–4), high (5–6) and very high risk (7–9) according to NIVO score

The performance of the NIVO score to predict NIV failure was better than its performance in predicting either in ICU mortality and 1-year mortality (AUC of 0.85) (Figure S1). In addition, patients with high or very high NIVO scores had significantly higher risk of NIV failure (HR of 11.8 [1.6–86.4] and 41.6 [5.6–307.9] respectively, plogrank < 0.0001) (Fig. 4A). A NIVO score ≥ 5 was associated with a 56% probability of NIV failure (Fig. 4B).

## Discussion

Our study findings indicate that the use of the NIVO score could be extended beyond short term (i.e. in-ICU) mortality prediction in patients with AECOPD requiring assisted ventilation. First, we found that its performance to predict 1 year mortality is acceptable even after excluding patients who died in the 30 days of admission. Second, the NIVO score showed good performance to predict early NIV failure in these patients. Higher NIVO score was strongly associated with early NIV failure.

The decision-making process in clinical practice, especially when considering patients with AECOPD, is often complex and influenced by various factors [18]. Thus, there is a clear demand for tools that can reliably predict mortality or distinguish between the success and failure of NIV. These tools should be based on objective and readily obtainable variables. Additionally, it is crucial for such a system to have the capacity to predict failure promptly [19], thereby preventing any delays in the decision to proceed with intubation or continue intensive care.

The NIVO score offers a straightforward calculation method requiring minimal information upon admission, making it potentially valuable for clinicians seeking a practical tool for prognostic assessment. However, there is currently limited evidence for supporting the use of the NIVO score in predicting mortality and guiding clinical decision-making for improved patient outcomes. Furthermore, the validity of this score developed in UK health care system remains to be determined abroad. In a cohort from China including 1184 patients with AECOPD [20], the NIVO score showed only moderate accuracy (AUC 0.66) to predict in-hospital mortality. In our cohort performed in a country outside UK, we confirmed the performance of NIVO-score to predict in-ICU mortality among patients with AECOPD requiring assisted ventilation. Indeed, AUC for predicting in-ICU mortality for NIVO score (0.79) was similar in our cohort than that of the index development study [7]. This may be explained by similarities among main characteristics in both cohorts. Indeed, demographics, severity of COPD (LTOT, FEV1), severity of the index exacerbation (PaCO_2_, pH) were similar between our cohort and those of the original NIVO score study. In addition, in-ICU mortality rate reported by Hartley et al. (25%) was in the same range with that of the current study (23%) [7].

Predicting 1 year mortality is also of foremost importance for decision-making in ICU in addition to short term prognosis. Indeed, among overall patients who were discharged alive from the ICU, 20% died over the following year [18, 21]. This is even more striking in patients with AECOPD for whom 1 year mortality is as high as 25% in those who were discharged from ICU [22]. Several scores have been developed to predict 1-year mortality in patients hospitalized in ICU and more specifically among patients with AECOPD [23, 24]. Nevertheless, the day-to-day use of these scores by clinicians in routine care remains uncertain. Furthermore, clinicians may be confused by the number of scores available and not know which one to use especially when some scores predict short term mortality and other mid-term mortality. In addition, clinicians need to understand and trust the tools they use. A simpler, more interpretable model might be preferred over a complex ensemble of scores, even if the latter offers a marginally better performance. Focusing on a few well-validated, interpretable, and practical predictive models is generally more effective for enhancing patient care and outcomes. We therefore aimed to explore additional outcomes of a well-developed and validated score. In the current study, the NIVO score’s ability to distinguish patients with a high risk of 1 year mortality was comparable to that of previously proposed scores [23]. Furthermore, after excluding patients who died within the 30 days of admission, the NIVO score at admission was still associated with mortality suggesting its use for predicting both short term and 1-year mortality.

Another challenge when admitting patients with AECOPD and AHRF is to appropriately initiate mechanical ventilation. NIV is an effective strategy for reducing mortality and invasive mechanical ventilation in patients with AECOPD and AHRF [25]. However, the NIV failure rate ranges from 20 to 50% in AECOPD [14, 17, 26]. In addition, patients who initially receive NIV but subsequently experience NIV failure are more likely to died. In the current study, NIV failure was of 37% and was associated with a 60% in-ICU mortality.

Identification of NIV failure is therefore of foremost importance. In the current study, we also explored the performance of the NIVO score to predict NIV failure. Regarding the association between higher NIVO scores and NIV failure in the group who received NIV, our findings reveal that patients with a score ≥ 5 on the scale had an 56% risk rate of NIV failure, which reaches 84% within the group with scores ≥ 7. The predictive performance of the NIVO score in identifying the risk of NIV failure was notably high in the current study. This may be explained by the strong relationship between NIV-failure and mortality among patients with AECOPD. Furthermore, the performance of NIVO score to predict NIV failure (AUC of 0.85) was similar to other previous prediction models such as the HACOR score (AUC of 0.83 in the external-validation cohort) [14]. Once again, the use of a single score to predict both mortality and NIV failure is probably more effective for enhancing patient care and outcomes.

Our study has several limitations. It was carried out in an ICU with an expertise in managing respiratory failure using NIV, equipped with suitable personnel and resources for patient care. Therefore, the generalizability of our findings to other units with varying skillsets, training, and infrastructure may be limited. Another limitation of our study is the relatively small sample size, which may have influenced the results and should be considered when interpreting our findings. Furthermore, since the study was conducted retrospectively, we were able to calculate the NIVO score for all patients included in our study; however, not all variables were available for collection and analysis, particularly when it comes to calculate other scores such as HACOR score for evaluating the risk of NIV failure and comparing it with the NIVO score.

## Conclusion

The NIVO-score is a reliable tool for predicting in-ICU mortality even in healthcare system outside the UK. Furthermore, our study suggests that the use of the NIVO score may be extended to predict 1-year mortality but also NIV failure with a good accuracy in AECOPD population.

## Supplementary Information

Supplementary Material 1.

## Data Availability

All data generated or analyzed during this study are included in this published article and its supplementary information files.
