# Supplementary material for: Predicting outcomes in patients with exacerbation of COPD requiring mechanical ventilation
Source: Ann Intensive Care. 2024 Oct 20;14:159. doi: 10.1186/s13613-024-01394-z (PMC11491423; doi:10.1186/s13613-024-01394-z)

Supplementary data

Table S1: ICD10-codes for identification of COPD patients, i.e., the source population. COPD: chronic obstructive pulmonary disease; ICD-10: International Classification of Diseases (revision 10), PD: primary diagnosis; SAD: second associated diagnosis.

ICD-10 codes	ICD-10 codes definition	Diagnosis in the summary of the hospital stay
**J44**	COPD	PD
**OR**		
**J44**	COPD	SAD and
**J09, J10, J11**	Influenzae	PD
**J12, J18, J20**	Respiratory infection	PD
**J93**	Pneumothorax	PD
**J960**	Acute respiratory failure	PD
**I50**	Acute heart failure	PD

Figure S1. Receiver operating characteristic (ROC) curves of NIVO score for NIV failure. Area under the ROC curve (AUC) values are also given.

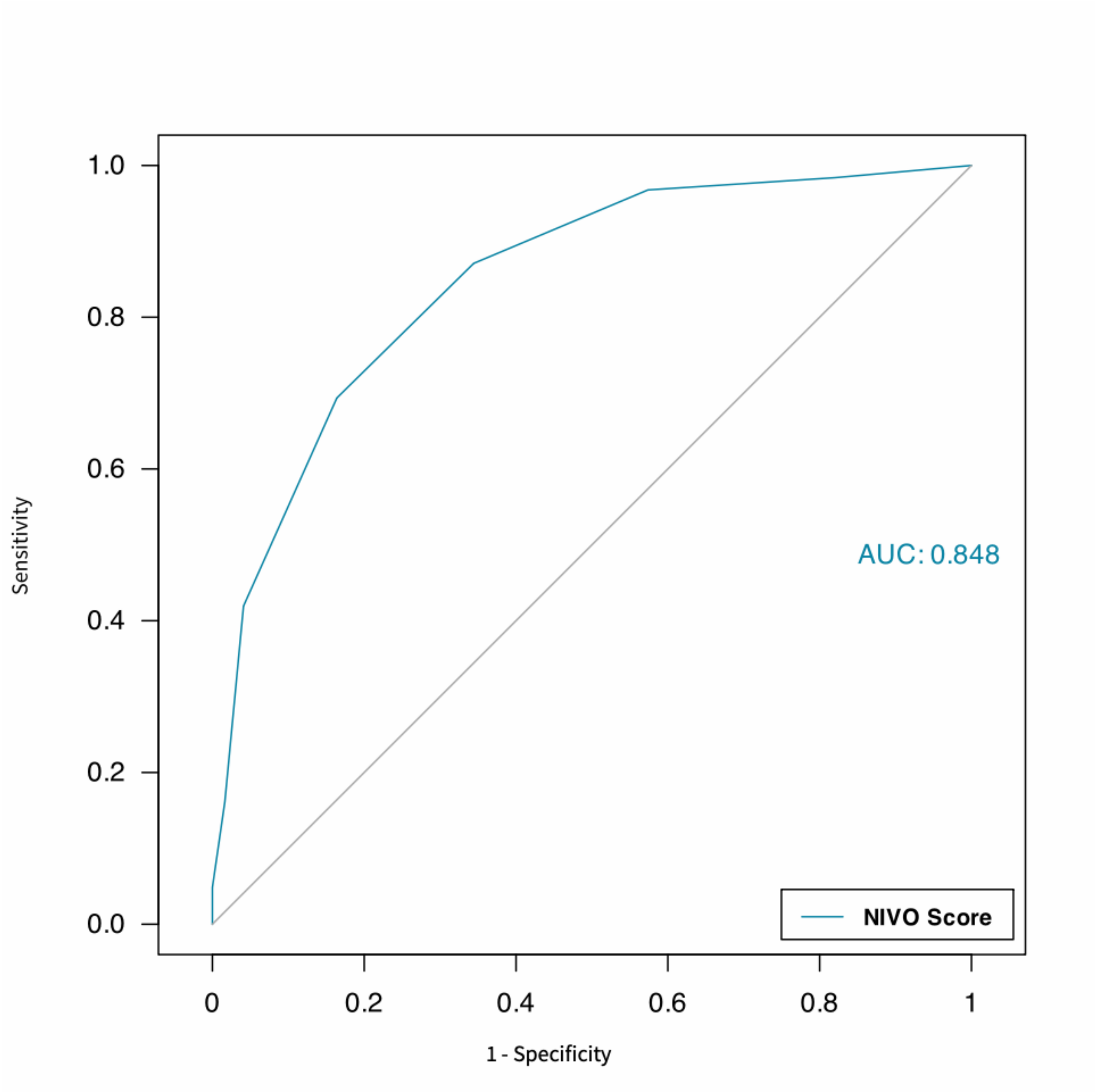

Supplement: Supplementary file 1 — Supplementary Material 1. [file 13613_2024_1394_MOESM1_ESM.pdf]
